# Limb salvage versus amputation: Experience of a young patient at a university hospital

**DOI:** 10.1016/j.tcr.2024.101062

**Published:** 2024-06-06

**Authors:** Juan Guillermo Ortiz Martínez, Edgar Manuel Bodu Lamberti, Pablo Ricardo Patarroyo Perea, Angela María Rico Avendaño, María Juliana Neira Barrero

**Affiliations:** University of La Sabana Clinic, Autopista Norte, Km 21, Chía, Colombia

**Keywords:** Amputation, Limb salvage, External fixator, Case report (MeSH)

## Abstract

Decision-making regarding limb amputation represents a significant clinical challenge, especially when the initial evaluation does not coincide with the criteria established in scales used worldwide, as is the case of the MESS scale. This article presents the case of a 24-year-old female patient who was transferred to a university hospital after a road traffic accident with severe and large lesions in the left lower limb. Despite a poor initial prognosis and in-hospital complications, including multiple surgical procedures and foot drop, a favorable recovery was achieved with complete anatomical salvage of the limb at risk. The multidisciplinary approach and intensive rehabilitation were instrumental in achieving a satisfactory functional recovery. This case highlights the importance of considering factors beyond amputation scale scores, as well as the need for comprehensive care to improve outcomes in patients with complex extremity injuries.

## Introduction

In 1990, Johansen and colleagues [1] proposed the usefulness of a scale with objective criteria for lower extremity amputation for which seven was the cut-off point for making an extreme decision, termed the Mangled Extremity Severity Score (MESS). We are frequently faced with highly complex patients in which the initial or admission clinical picture does not correlate with the appropriate or necessary score for making a decision on limb amputation [2,3]. We present the case of a young female patient, with no past medical history, in which despite the complexity of the lesions, poor prognosis of the limb and continued postoperative dysfunction, had a favorable in-hospital recovery and anatomical salvage of the limb.

## Case history

**Fig. 1:**
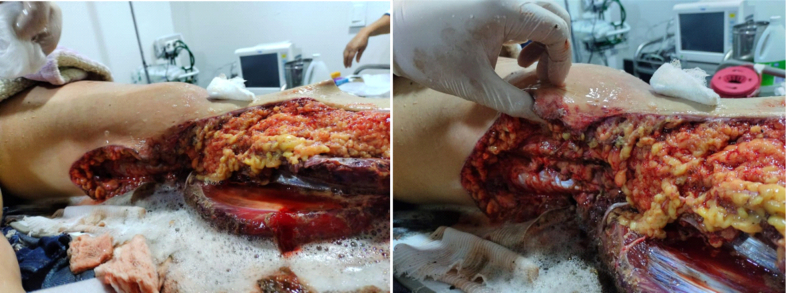
Circumferential degloving lesion of the left leg with extension to the posteromedial thigh and muscle exposure associated with macro contamination.

A 24-year-old female patient with no past medical history was admitted to the emergency department after inter-facility transfer due to a high energy road traffic accident as a motorcycle passenger colliding with an automobile presenting trauma to the left lower limb. Vital signs on admission were as follows: blood pressure 95/54 mmHg and mean arterial pressure 68 mmHg, heart rate of 125 beats per minute, respiratory rate of 22 breaths per minute with a Glasgow Consciousness Score of 14/15. On primary evaluation, the airway was patent, with no lesions on the face or neck. Similarly, the thorax was symmetrical in its expansion; FAST ultrasound was performed within normal parameters. Regarding her hemodynamic status, the patient went into hemorrhagic shock class II requiring transfusion of two units of packed red blood cells and administration of one gram of tranexamic acid. A long leg splint was evidenced in the left lower limb which was removed observing a large degloving lesion (Morel-Lavallée) of the leg with extension to the posteromedial thigh (Fig. 1). No alterations in distal perfusion or other open injuries were observed.

An extra-institutional leg x-ray was reviewed and showed a comminuted diaphyseal fracture of the tibia and fibula with debris in the adjacent tissues. Therefore, it was considered that the patient sustained an open fracture of the left tibia and fibula Gustilo and Anderson (GA) IIIB and a macerated extremity (MESS six points) secondary to polytrauma with an Injury Severity Score (ISS) of 29 points, thus initiating antibiotic coverage with cefazolin, penicillin, gentamicin and is taken to surgery immediately for debridement and external fixation.

**Fig. 2:**
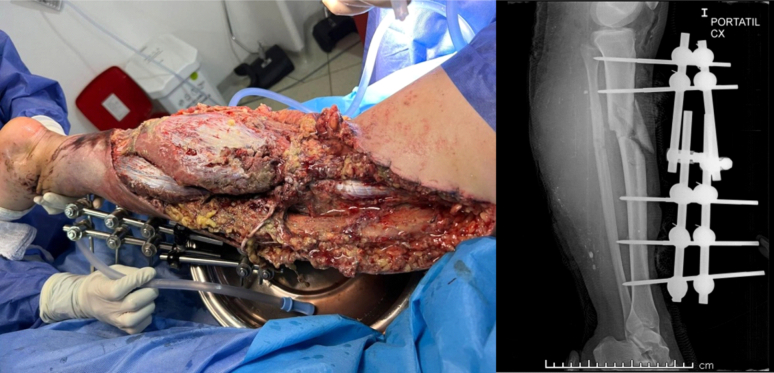
External fixator, lateral gastrocnemius vicinity flap and postoperative x-ray with acceptable fragment alignment.

During the first surgical intervention, there was intraoperative evidence of great contamination due to the presence of organic debris (rocks, soil, asphalt), injury to the extensor tendons of the foot and ankle, partial injury of the lateral gastrocnemius, posterior tibial muscle injury and hamstring injury without active arterial bleeding. A vicinity flap of the lateral gastrocnemius was advanced to achieve adequate coverage; the tibia was stabilized with a modular external fixator and application of negative sub-atmospheric pressure system (VAC®) was done (Fig. 2). She was subsequently admitted to the intensive care unit without ventilatory or vasopressor support for neurological monitoring due to a skull base fracture evidenced in the cranial computed axial tomography during the secondary evaluation.

**Fig. 3:**
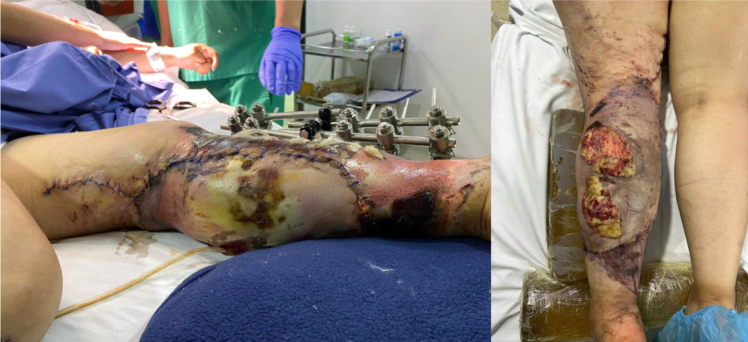
Partial skin grafts applied in thigh, popliteal fossa and leg.

After four days, the patient was transferred to the hospitalization floor and required multiple washings and debridements due to the large macro contamination of the tissues, periodically evaluating the integumentary defects of the leg accompanied by the plastic surgery department to define the possibility of coverage. Given the presence of fetid serous secretion, intraoperative cultures were taken and reported negative after 72 h of incubation; for this reason, partial skin grafts were performed by the plastic surgery department three weeks later, once infection control was achieved (Fig. 3), initiating the multidisciplinary reconstructive process.

**Fig. 4:**
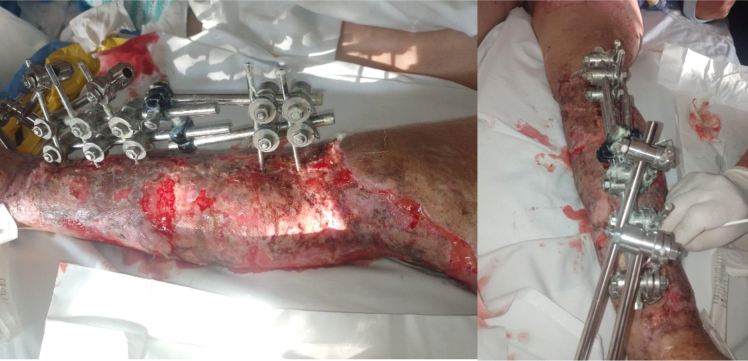
Multiplane external fixator in leg with extension to the calcaneus, integration and epithelialization of skin grafts.

In order to ensure plasma imbibition of the grafts, wound cleaning was done every five days, regularly evaluating skin condition to define the appropriate surgical time for the application of a reconstructive (multiplane) external fixator; owing to post-traumatic foot drop caused by injury of the extensor tendons of the foot and ankle, peroneal nerve injury as well as severe retraction of the Achilles tendon, preventing dorsiflexion. The grafts presented an adequate integration and five weeks after starting the reconstructive process, the patient was taken to an Achilles tenotomy and rearrangement of the external fixator, positioning the Schanz pins at the calcaneus, stabilizing the foot in dorsiflexion so that the patient could stand upright (Fig. 4). Regarding the definitive management of the tibia fracture, the presence of foot drop would impede the rehabilitation process if intramedullary nailing was decided, since one of the principles for secondary bone consolidation is early weight bearing. For this reason, a reconstructive external fixator with the foot fixed in dorsiflexion was left as the definitive management.

**Fig. 5:**
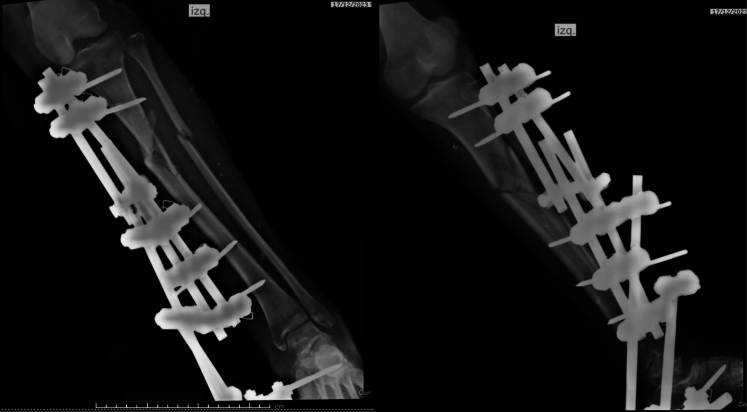
Anteroposterior (AP) and lateral x-ray of the left leg showing external fixator and satisfactory alignment.

After showing clinical improvement, the patient began a rehabilitation process with physiatry, focused on physical therapy and muscle strengthening. Gradual progress was made in training, advancing from sitting to standing with monopodal support and external assistance using a walker. Leg x-rays were taken one month postoperatively after rearrangement of the external fixator to evaluate bone consolidation, observing satisfactory alignment (Fig. 5). Finally, she was discharged three months after her initial admission under a home hospitalization plan consisting of wound care and physical therapy, returning to her city of origin.

**Fig. 6:**
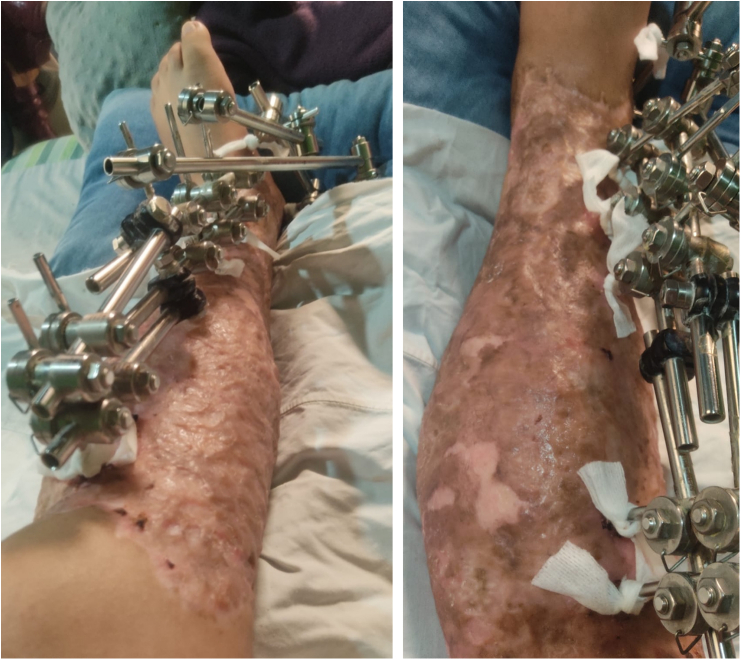
Full epithelialization of skin grafts, no signs of infection at pin sights.

**Fig. 7:**
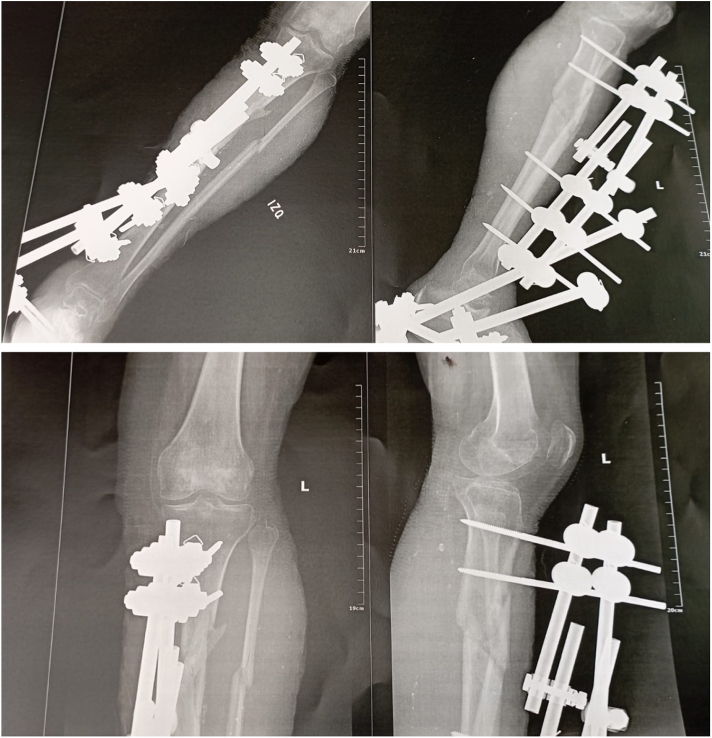
AP and lateral x-ray of the left leg and knee observing callus formation of the tibia and fibula.

The patient was seen five months later by teleconsultation observing full epithelialization of the skin grafts and callus formation of the tibia and fibula (Fig. 6, Fig. 7). Health related quality of life was quantified by the SF-12 questionnaire scoring 40.42 in the physical component and 55.24 in the mental component, indicating a state of well-being and functional recovery of the limb.

## Discussion

Therapeutic advances in limb salvage have called into question the validity of the scoring systems used to choose ablative or reconstructive management of the limb. Clinical decision making should be based on an individualized approach to ensure the best physical, functional, psychological and socioeconomic outcomes for the patient. In the LEAP (Lower Extremity Amputation Project) study, Bosse and colleagues [4] evaluated the clinical utility of five scales applied in severe lower limb injuries, observing a sensitivity of 8 % and specificity of 94 % in the MESS scale in GA IIIB open tibia fractures, suggesting that it would delay the identification of those patients who will eventually require amputation, which increases the risk of complications [5].

Schirò and colleagues [6] conducted a systematic review with the aim of evaluating the most commonly used scales to guide the management of the macerated limb, finding controversial results in which they report high rates of primary amputation (14.5 %) in patients with a MESS score of less than seven. Due to the lack of consensus on the use of these tools, strategies need to be developed to provide rapid and assertive care.

In this case, the MESS scale was applied and a score of six was obtained, indicating preservation of the affected limb. However, the torpid evolution during her hospital stay due to multiple surgical interventions and complications, such as post-traumatic foot drop that did not allow standing, led to consider amputation as a therapeutic alternative. However, multiple surgical efforts were made using an orthoplastic management that was decisive in saving the limb. In a systematic review by Klifto and colleagues [7], they observed how orthoplastic management provides a synergistic model to optimize and accelerate definitive skeletal fixation and soft tissue coverage, as was performed in the patient's case. Although definitive management with a multiplane external fixator is not the standard of care in tibial shaft fractures, it allowed for early weight bearing which was fundamental for rehabilitation. The FIXIT study [8] aimed to evaluate the one-year probability of a major limb complication in patients with an open tibial shaft fracture treated with either internal fixation or modern external ring fixation, observing no appreciable differences in the probabilities of amputation, nonunion, soft-tissue problems, malunion, or fracture healing between the groups.

Zeiderman and colleagues [9] carried out a review article on the reconstruction of the lower limb after trauma where they mention the factors to define limb salvage: young patient, absence of tibial nerve injury, no signs of ischemia and a good rehabilitation potential; indicating that clinical decision making in these patients should not rely solely on evaluation scales.

The MESS scale is a helpful predictor when deciding on an ablative or conservative approach to complex extremity injuries, taking into account the patient's comorbidities and rehabilitation potential. Diverse results have been found in the literature regarding its application and usefulness, for which additional tools and strategies are needed to correctly identify patients who are candidates for limb salvage.

## Informed consent

The patient signed an informed consent form authorizing the treatment of her medical record as well as the images obtained in this report.

## Financing

None stated by the authors.

## CRediT authorship contribution statement

**Juan Guillermo Ortiz Martínez:** Writing – review & editing. **Edgar Manuel Bodu Lamberti:** Writing – review & editing, Writing – original draft. **Pablo Ricardo Patarroyo Perea:** Writing – original draft. **Angela María Rico Avendaño:** Writing – original draft. **María Juliana Neira Barrero:** Writing – original draft.

## Declaration of competing interest

None stated by the authors.

## References

[1] Johansen, Kaj M.D., Ph.D.; Daines, Michael M.D.; Howey, Thomas M.D.; Helfet David M.D.; Hansen, Sigvard T. Jr. M.D.. Objective criteria accurately predict amputation following lower extremity trauma. The Journal of Trauma: Injury, Infection, and Critical Care 30(5):p 568–573, May 1990. 10.1097/00005373-199005000-00007 2342140

[2] Loja, Melissa N. MD, MAS; Sammann, Amanda MD, MPH; DuBose, Joseph MD; Li, Chin-Shang PhD; Liu, Yu MS; Savage, Stephanie MD, MS; Scalea, Thomas MD; Holcomb, John B. MD; Rasmussen, Todd E. MD; Knudson, M. Margaret MD The AAST PROOVIT Study Group. The mangled extremity score and amputation: time for a revision. Journal of Trauma and Acute Care Surgery 82(3):p 518–523, March 2017. | DOI: 10.1097/TA.0000000000001339. PMC5321791 28030489

[3] Gratl A., Kluckner M., Gruber L., Klocker J., Wipper S., Enzmann F.K. (Dec 2023). The mangled extremity severity score (MESS) does not predict amputation in popliteal artery injury. Eur. J. Trauma Emerg. Surg..

[4] Bosse M.J., MacKenzie E.J., Kellam J.F., Burgess A.R., Webb L.X., Swiontkowski M.F., Sanders R.W., Jones A.L., McAndrew M.P., Patterson B.M., McCarthy M.L. (Jan 1 2001). A prospective evaluation of the clinical utility of the lower-extremity injury-severity scores. JBJS.

[5] Kauvar, David S. MD; Thomas, Sarah B. MD; Schechtman, David W. MD; Walters, Thomas J. PhD. Predictors and timing of amputations in military lower extremity trauma with arterial injury. Journal of Trauma and Acute Care Surgery 87(1S):p S172-S177, July 2019. | DOI: 10.1097/TA.0000000000002185. 31246923

[6] Schirò G.R., Sessa S., Piccioli A., Maccauro G. (Dec 2 2015). Primary amputation vs limb salvage in mangled extremity: a systematic review of the current scoring system. BMC Musculoskelet. Disord..

[7] Klifto K.M., Azoury S.C., Othman S., Klifto C.S., Levin L.S., Kovach S.J. (Mar 22 2021). The value of an orthoplastic approach to management of lower extremity trauma: systematic review and meta-analysis. Plast. Reconstr. Surg. Glob. Open.

[8] Major Extremity Trauma Research Consortium (METRC)*. Modern external ring fixation versus internal fixation for treatment of severe open tibial fractures: a randomized clinical trial (FIXIT Study). The Journal of Bone and Joint Surgery 104(12):p 1061–1067, June 15, 2022. | DOI: 10.2106/JBJS.21.01126. 36149241

[9] Zeiderman M.R., Pu L.L. (2021). Contemporary approach to soft-tissue reconstruction of the lower extremity after trauma. Burns & Trauma..

